# Subjective Well-being during the Pandemic: A Pilot Study in the Cuban Population

**DOI:** 10.11621/pir.2021.0308

**Published:** 2021-09-30

**Authors:** Evelyn Fernández-Castillo, Diana Rosa Rodríguez-González, Zoylen Fernández-Fleites, Yunier Broche-Pérez, Idania María Otero-Ramos, Lesnay Martínez-Rodríguez, Annia Esther Vizcaíno-Escobar, Reinier Martín-González, Dunia Mercedes Ferrer- Lozano, Ellis Elaine Palmero-Betancourt

**Affiliations:** a Psychology Department, Universidad Central “Marta Abreu” de Las Villas, Santa Clara, Cuba; b University Well-being Center, Santa Clara, Cuba; c CognitiON (Cuban Iniciative on Cognitive Health), Santa Clara, Cuba; d Community Studies Center, Hamilton, Canada; e Palmero Counselling, Santa Clara, Cuba

**Keywords:** Subjective well-being, gender, COVID-19, psychological impact, Cuban population

## Abstract

**Background:**

The study of aspects related to positive mental health and well-being in the general population with a gender approach is a necessity in the current context imposed by COVID-19.

**Objective:**

To explore gender as a predictor of subjective well-being during COVID-19 in a sample of the Cuban population.

**Design:**

A cross-sectional web-based survey design was adopted. The sample consisted of 129 Cuban participants. The Subjective Well-being-Reduced Scale (SW-RS) was used to explore subjective well-being in the sample. Descriptive statistics were used to explore the participants’ characteristics. A multinomial logistic regression analysis was conducted to identify variables independently associated with the participants’ subjective well-being.

**Results:**

The gender of participants significantly predicted subjective well-being levels. The probability of males having middle or high levels of subjective well-being, rather than low levels, was 4.16 times greater than in females. The probability of males having a high self-image instead of a low one was 4.35 times greater than in females. According to the *self-satisfaction* dimension, the odds of males having high rather than low self-satisfaction were five times more than in females. In this sample, gender did not predict whether participants had middle or high levels of the hedonic dimension.

**Conclusion:**

Our results corroborated international studies that have indicated the coincidence of lower well-being and greater psychosocial risk in women during the COVID-19 pandemic. The results also indicated the need to dig deeper into the experiences of subjective well-being from a gender perspective, and to strengthen the sufficiency and effectiveness of the actions and guidance that are offered to the population from psychological care services, the media, and public policies.

## Introduction

In recent decades, there has been a change in the field of mental health from being focused on pathologies, discomfort, and finding ways to provide interventions aimed at improvement, to focusing on the absence of disorders and the promotion of wellbeing (Martínez, 2021). Thus, well-being studies currently occupy a large part of the work done in the field of psychology. There are several perspectives related to the study of well-being (Vielma & Alonso, 2010); despite this, there is general agreement that well-being is a multivariate construct of a stable nature in mobile equilibrium (Zotova & Karapetyan, 2018).

The dominant perspectives on the study of well-being are the hedonistic tradition, which focuses on happiness, the presence of positive affect, and the absence of negative affect (Deci & Ryan, 2008), and the eudaimonic tradition, which focuses on living life to its fullest and realizing one’s human potential (Diener, 1984). Both perspectives consider happiness and well-being as indicators of the degree to which a person is psychologically fulfilled; however, each perspective has foundations that reflect very subtle differences in their points of view. The hedonic perspective of wellbeing varies from a relatively limited focus on bodily pleasures to a broad focus on personal interests. This model focuses mainly on the results of happiness or pleasure, and stresses the perspective that well-being consists of subjective happiness, which includes as primary components satisfaction with life, the presence of a positive mood, and the absence of negative mood (Ryan & Deci, 2001).

From the eudaimonic perspective, Diener’s model of subjective well-being is the one most supported by the scientific community and is shared by the authors of this article. Diener highlights the role that cognitive and affective evaluations play in a person’s life. These evaluations include emotional reactions to events and judgments about satisfaction and achievement. This concept includes the experience of pleasant emotions, a low level of negative emotions, and a high level of satisfaction with life (Diener, 2000).

Scientific interest in the role that subjective well-being plays in an individual’s development has led to numerous follow-on research projects. These studies include ones that have shown the effect of positive or negative events on the individual’s subjective well-being . The meta-analysis carried out by Koydemir et al. (2020), with non-clinical adult populations, showed that positive psychological interventions increase subjective well-being. Other research has emphasized the relationship between various sociodemographic variables and subjective well-being. For example, it has been found that there are changes in subjective well-being as age advances, with a progressive decrease from early to late adolescence, and then again from 60 years of age on (Casas et al., 2012; Wunder et al., 2013).

Studies on the gender variable and subjective well-being have not been totally conclusive. The European Union’s publication *Quality of Life: Facts and Views* (2015) stated that women reported greater satisfaction with life than men; however, they also showed a greater tendency for depressive symptoms. Senik (2015) affrmed a gender gap that varies throughout life, while Valls-Llobet (2009) asserted that gender is the foundation for determining the individual’s state of health or disease. For these authors, gender differences were associated with differences in the prevalence of certain disorders, in the response to treatment alternatives, and in the attitude taken towards care. On the other hand, Hyde (2005) maintained that the similarities between genders and subjective well-being were greater than the differences.

In Cuba, gender inequalities are much lower than those of other Third World countries in the region. However, studies have revealed the presence of gender inequities, especially in everyday life, which could mark differences in the well-being of men and women. In recent decades, some studies have shown differences between men and women in terms of sexual and reproductive health, rest time, the perception of diseases or symptoms, and the search for medical attention (Castañeda Abascal et al., 2010). Researchers have warned of inequity gaps in the distribution of caregiving tasks, with a disproportionate burden and costs to the health of females (Romero, 2019). Different studies have also placed women within the groups most likely to suffer different forms of violence and the burden of tasks within the family, which affects their physical and emotional health (Ferrer Lozano et al. 2020), while Fleitas (2013) has shown the close relationship between gender, poverty, and health, and identified women as the most affected group.

### Effects of the Pandemic on Well-being

There is considerable consensus when it comes to the role that life events play as determinants of well-being. Negative and stressful events have been associated with the presence of a negative subjective state of well-being (Argyle, 2001). As the pandemic has worsened on a global scale, and forced self-care, confinement at home, and changes in lifestyle, studies have pointed to a greater incidence of emotional disorders such as depression, stress, apathy, irritability, insomnia, post-traumatic stress disorder, anger, and emotional exhaustion (Broche-Pérez, Fernández-Castillo, & Reyes, 2020), evidencing the negative impact that the pandemic has had on the well-being of the population (Boukhris et al., 2020). For example, in a study conducted in Australia, it was found that there was a decrease in the population’s subjective well-being due to the social and workplace difficulties that resulted from the pandemic (Dawel et al., 2020). In particular, the researchers reported a significant increase in depressive and anxious symptoms among Australians.

In Germany, Zacher and Rudolph (2020) conducted a study on well-being in the general population. The results indicated that, during the months between December 2019 and March 2020, the pandemic did not significantly affect the well-being of people in Germany. However, between the months of March and May of 2020, the psychological well-being of Germans was significantly affected. O’Connor et al. (2020) reported an increase in suicidal ideation in the general population during the first wave of the pandemic in the UK, especially among young adults.

In this sense, it is important to understand how some variables modulate the impact of the current pandemic on the subjective well-being of the population. One of these variables is gender, and thus the link between gender and well-being has been studied by several authors during the current epidemiological situation.

During the current pandemic, there have been differences reported between the levels of happiness of men and women (Gausman & Langer, 2020; Yildirim et al., 2020). In general, women have reported a lower level of psychological well-being, related to the biological, psychological, and environmental factors that typically affect them. For example, in a longitudinal study conducted in Spain to explore gender-related differences in the psychological impact of COVID-19 lockdowns, clear differences were found between the well-being of women and men, with women showing significantly lower levels of well-being (Ausín et al., 2021). Also, in a study that looked at the mental health and psychological well-being of the UK adult population during the first six weeks of the COVID-19 pandemic, men reported significantly higher levels of psychological well-being than women (O’Connor et al., 2020).

In Cuba, studies on the impact of COVID-19 on the mental health of the population have shown that women presented with higher levels of fear (Broche-Pérez et al., 2021). These findings showed that fear of COVID-19 was more severe in female participants than male ones, and that gender was a predictor of the level of fear of COVID-19 (Broche-Pérez et al., 2020).

However, there are still a limited number of studies in our context that explore aspects related to positive mental health in the general population, and that delve into the impact of COVID-19, with a focus on gender. Distinguishing gender when it comes to well-being could guide future preventative actions, helping to make them more effective. The objective of our study was to explore gender as a predictor of psychological well-being during the COVID-19 pandemic in a sample of the Cuban population.

## Methods

A cross-sectional web-based survey design was adopted.

### Participants

Participants completed a survey via Google Forms®, between April 14 and August, 2020. All Cuban citizens over the age of 18 were eligible to participate in the study. Nonprobability samples were used. Snowball sampling was conducted to recruit participants. The survey was disseminated through WhatsApp groups, Facebook, email lists, and the website of the Well-being Center of the Universidad Central “Marta Abreu” de Las Villas.

A total of 129 people participated in the study (72.1% females and 27.9% males); the mean age was 36.4 years old (SD 12.3). Online consent was obtained from the participants.

### Procedure

All subjects were asked to fill out the Subjective Well-being-Reduced Scale (SW-RS) questionnaire: We used the scale adapted by Torres-Acuña (2003). Its abbreviated version was validated for the Cuban population and showed acceptable psychometric properties (α = .70). The results of the exploratory factor analysis performed on the scale showed a structure of four factors with acceptable reliability indices.

Factor 1 (F1) was associated with the hedonic aspects of well-being, such as leisure time, fun, and socialization (*i.e.,* “I like to have fun”). The second factor (F2) explored the dimension of satisfaction with life (*i.e.,* “I think that I have achieved what I wanted as a person”). The third factor (F3) explored satisfaction with daily activities, sense of purpose, and the eudaimonic dimension of well-being (*i.e,* “I face my tasks with a good mood”). The fourth factor (F4) grouped items on which the individual reports a self-image of being healthy, through a positive perception of their mood (*i.e.,* “I think I have good health”). Each item was answered using a Likert scale as follows: 1 = Never or almost never; 2 = Sometimes; 3 = Very often; 4 = Almost always; and 5 = Always. (Rodríguez-Martín et al., 2012).

In the present study, the scale showed acceptable reliability indices both in general (α = .878) as well as for each of its four dimensions (F1 α = .745; F2 α = .758; F3 α = .796; F4 α = .512).

#### Data Analysis

The study was approved by the ethics committee of the Department of Psychology of the Universidad Central “Marta Abreu” de Las Villas. Informed consent to be included in the study was obtained from all participants. Participation was voluntary, and the anonymity of the participants was guaranteed.

The data was processed using SPSS v21.0. Descriptive statistics were used to explore the participants’ characteristics. Logistic regression (LR) was the best statistical choice for processing the data, as it took two main criteria into account: 1) the type of sample and the non-parametric design of the data analysis, and 2) the practical value of the results obtained through LR in the current epidemiological situation. Scores on the well-being scale were grouped by levels (high, medium, and low); the results allowed for the detection of people who might be at-risk, taking the predictor variables as a reference.

LR allows the test of models which can predict categorical outcomes with two or more categories. The independent variables can be either categorical or continuous, or a mix of both (Field, 2009, 2012; Pallant, 2005). A model of the main effects for multinomial logistic regression was constructed to identify variables independently associated with well-being. The three subjective wellness levels were established according to the method established by Rodríguez-Martín, Molerio, Castillo, and Pedraza (2012). These authors describe three levels for each factor as well as for the global variable. The threshold of statistical significance used in this study was p < 0.05.

## Results

### Respondent Characteristics

The characteristics of the respondents are shown in *Table 1.* The larger proportion of respondents were women (72.1%). The mean age was 36.47 years (SD ± 13.01), and most participants were in the 29-41 age group (34.31%). Boundaries of age groups were established according to sample percentiles. Age was measured in all three categories, to put in the middle the values of SD, minimum and maximum only respond to spatial location.

**Table 1:** Demographic characteristic of the sample

		*fr* **(%)**	M(SD)	Min.	Max.
Gender	Male	36(27.9)			
	Female	93(72.1)			
Age groups	Until 28	34(26.4)			
	29–41	59(45.7)	36.4(12.3)	18	76
	More than 42	36(27.9)			

*Note. M = Mean. SD = Standard Deviation. fr = Frequency.*

Results from the SW-RS are shown in *Table 2.* Global well-being scores registered a mean of 37.75 (SD ± 7.30). The middle level of well-being showed the highest frequencies for F4 (M = 7.44, SD ± 1.77, 55.0%) and F1 (M = 10.82, SD ± 2.02, 48.1%), as well as for the global score (49.6%). The highest level of subjective well-being occurred in the “Self-satisfaction” dimension, with a score of 32.6% (M = 10.87, SD ± 2.73).

**Table 2:** Results from SW-RS score and levels in the sample

						Levels *fr*(%)	
	Median	SD	Min.	Max.	Low	Middle	High
Well-being	37.75	7.30	10	50	36(27.9)	64(49.6)	29(22.5)
F1 Hedonics	10.82	2.02	4	15	54(41.9)	62(48.1)	13(10.1)
F2 Self-satisfaction	10.87	2.73	3	15	34(26.4)	53(41.1)	42(32.6)
F3 Activities performed	6.89	1.37	3	10	87(67.4)	41(31.8)	1(.8)
F4 Healthy self-image	7.44	1.77	3	10	43(33.3)	71(55.0)	15(11.6)

*Note. SD = Standard Deviation. fr = Frequency.*

### Association between Gender of Participants and Subjective Well-being

Gender was investigated using Multinomial logistic regression (LR). Demographic was explored as a demographic factor independently associated with levels of subjective well-being. The factor *Activities performed* was not included in the LR because of the existence of empty cells in the expected frequency. There were two cells (33.3%) with frequencies lower than 5, so, according to the standard set by Pallant (2005), that factor was not included in the LR. Preliminary analyses were performed to ensure no violation of the assumptions of linearity, independence of errors, and multicollinearity (Field, 2012). *Table 3* shows final analysis was conducted on a total of 129 valid cases.

**Table 3:** Demographic and social factors independently associated with well-being during COVID-19 pandemic

	Global Well-being				Healthy Self-image				Self-satisfaction				Hedonic			
	95% CI				95% CI				95% CI				95% CI			
	B(SE)	Inf.	OR	Sup.	B(SE)	Inf.	OR	Sup.	B(SE)	Inf.	OR	Sup.	B(SE)	Inf.	OR	Sup.
Middle vs. Low Well-being^a^																
Intercept	1.71(.54)				.75(.41)				1.32(.56)				.25(.36)			
Female	–1.43(.59)*	.08	.24	.76	–.32(.46)	.29	.72	1.79	–1.09(.61)	.10	.34	1.12	–.16(.42)	.38	.86	1.94
High vs. Low Well-being^a^																
Intercept	.92(.59)				–.12(.49)				1.45(.56)				–1.25(.57)			
Female	–1.44(.66)*	.07	.24	.86	–1.46(.64)*	.07	.23	.81	–1.63(.62)**	.06	.20	.66	–.24(.68)	.21	.79	2.97

Note. CI = Confidence Intervals. ^a^ = Reference Category. * p < 0.05; ** p < 0.01; *** p < 0.001.

Gender predicted both whether participants had middle or low global well-being during the Coronavirus pandemic [b = –1.43, Wald X2(1) = 5.86, p < 0.05], and whether they had high or low levels of well-being [b = –1.44, Wald X2(1) = 4.76, *p < 0.05]. In both cases, comparing the effect on females with that on males, the odds ratio indicated that as the variable changed, the change in the odds of having middle or high levels of well-being instead of low levels were .24. The odds that males had middle or high levels of well-being rather than low levels was 1/.24, or 4.16 times greater than for females.

Gender also significantly predicted whether people had a high or low healthy self-image [b = –1.46, Wald X2(1) = 5.24, p < 0.05] and self-satisfaction [b = –1.63, Wald X2(1) = 6.95, p < 0.01]. The odds of males having a high self-image instead of a low one were 1/.23, or 4.35 times greater than in females. According to the self-satisfaction dimension, the odds that males had high rather than low levels of self-satisfaction were 1/.20, or 5 times more than females.

In the sample, gender did not significantly predict whether participants had middle or high levels in the hedonic dimension.

## Discussion

The objective of this study was to explore gender as a predictor of subjective well-being during COVID-19 in a sample of the Cuban population. Our results showed that the gender of participants did significantly predict the level of subjective well-being in a sample of this population.

Being male was a predictor of medium to high levels of subjective well-being during COVID-19. Gender also significantly predicted whether people had a high or low healthy self-image and self-satisfaction. Furthermore, gender did not significantly predict whether participants had medium or high levels on the hedonic dimension. These results confirmed the findings of previous studies. For example, in a study conducted by Yildirim et al. (2020) on 4,536 Turkish adults, the authors examined the effects of vulnerability, perceived risk, and fear on COVID-19 preventive behaviors. The authors reported that women were more vulnerable than men to experiencing fear.

On the other hand, Burdett et al. (2020) reported that the decline of women’s mental health in the UK was twice that reported by men. In Germany, a greater decline in the psychological well-being of women than of men due to COVID-19 has been reported. The growth of the negative impact of the pandemic on women’s psychological well-being corresponded to the progression of the disease in that country (Mutz, 2020).

In a qualitative study carried out in Argentina, women showed greater intensity in their reactions to fear and anguish. Women also showed a greater level of responsibility in self-care, and were more self-reflective about the consequences of the pandemic (Johnson et al., 2020).

Similar results were found by Hill et al. (2016) in the different dimensions they studied; their results showed that gender had a strong effect on self-image. A meta-analysis by Gentile et al. (2009) found that men scored significantly higher than women on self-satisfaction (d = 0.33). This meta-analysis examined gender differences in 10 specific domains of self-esteem across 115 studies, including 428 effect sizes and 32,486 individuals.

In general, women tend to experience more negative affect, and their subjective well-being deteriorates more than men’s. However, women also show more adaptive ways of coping with stress and tend to manage their emotions better. This is a tentative explanation as to why in the present study gender did not predict medium and high levels of the hedonic dimension of subjective well-being. In this regard, LeFebvre & Huta (2021) only found a significant difference in the motivation for hedonic pleasure between men and women in subjects 20 to 29 years old (sample: 701 women and 623 men, ages 18-87). LeFebvre and Huta (2021) did not find differences in the motivation for hedonic comfort.

The COVID-19 crisis has strengthened the differences between men and women in terms of mental health and well-being, although this had already been happening. Pre-pandemic studies found that men are less likely to suffer from common mental health disorders than women (Shepherd, 2016). Xu and Wang (2021) found that older women have more abundant and meaningful activities of daily living and receive more emotional support than men, and that these differences are related to their gender roles.

To understand the results found in this study of subjective well-being from a gender perspective, it is important to understand that it is a socially constructed category, and take into account its strong cultural variation among different countries (Álvarez, 2014). During socialization, emotional expression and caretaker roles are encouraged in women, while men are often told that they should be “strong” and should not show their emotions (Álvarez, 2015).

On the other hand, there are studies that suggest that the differences in subjective well-being between women and men are due to the intensity of their emotions, not the frequency with which these emotions are experienced. Reviews of gender differences in subjective well-being consistently agree that women tend to experience higher levels of unpleasant affect than men, but that these differences are limited to internalizing moods. Women report more frequent and intense internalized moods, such as sadness, fear, nervousness, shame, and guilt. In general, women tend to report more intense and frequent negative, unpleasant, and internalized emotions than men (Lucas & Gohm, 2000).

In summary, to explain the variability reported during the pandemic in the mental health indicators of both genders, multiple factors must be considered. Some factors predate the pandemic (such as the social construction of gender), and other factors are specific to this period of time (such as the burden of the caregiver role and remote work). These results must be taken into account when designing actions aimed at enhancing the subjective well-being of Cuban women.

## Conclusion

In conclusion, our findings showed that the gender of participants significantly predicted the level of subjective well-being in a sample of the Cuban population. The results corroborated research carried out in other contexts where it was suggested that women have lower levels of well-being, which could in turn be associated with greater psychosocial risk during the current COVID-19 pandemic.

The results emphasized the importance of considering gender differences when designing interventions aimed at improving subjective well-being. In addition, our study offers initial results that could be useful in the design of actions aimed at promoting health.

## Limitations

This research is not without limitations. First, the sample for this preliminary study is small. In future studies, the sample size should be increased to obtain more generalizable results. Furthermore, it is a cross-sectional study, so it is difficult to capture the causal relationships between gender and subjective well-being. It is important to develop longitudinal studies that explore the relationship between both of these variables and other variables that could be mediating this relationship at different times during the pandemic.
